# Spontaneous trans-anal extrusion of caudally migrated ventriculo-peritoneal shunt tip in a child: a case report

**DOI:** 10.1186/s40792-020-00813-0

**Published:** 2020-03-10

**Authors:** Sabyasachi Bakshi

**Affiliations:** 1 IPGMER & SSKMH, Kolkata, West Bengal PIN-700020 India; 2 Kathghara Lane, Sonatuli, Hooghly, West Bengal 712103 India

**Keywords:** Trans-anal extrusion, VP shunt, Hydrocephalus

## Abstract

**Background:**

Bowel perforation caused by the ventriculo-peritoneal shunt is a rare occurrence with an estimated incidence rate of 0.1% to 1.0% among all cases of VP shunt displacement. This is an unusual report of spontaneous trans-anal extrusion of caudally migrated ventriculo-peritoneal shunt tip in a child. Literature was reviewed to find out therapeutic strategies.

**Case presentation:**

An asymptomatic 8 months old boy presented with spontaneous trans-anal extrusion of caudally migrated left-sided Chhabra type of ventriculo-peritoneal (VP) shunt for last 1 day, following surgery for hydrocephalus initially done 3 months ago. He had no features of peritonitis or encephalitis. Digital X-ray of the whole abdomen in postero-anterior view in erect posture was only evident of the expulsion of radio-opaque distal catheter tip through the anus into the exterior. Noncontrast-enhanced computed tomography scan (NCCT) of brain showed proximal catheter in the lateral ventricle of the brain. Under sedation, the distal part of the VP shunt catheter was resected out, aseptically, over the abdomen and pulled out gently through the anus. The proximal catheter part along with the reservoir was removed through a separate incision in the neck and sent for bacteriological study, which came out later to be negative. Postoperatively, the child was put on a prophylactic antibiotic and 3 weeks later another VP shunt was placed in the contralateral side.

**Conclusions:**

Spontaneous trans-anal extrusion of VP shunt tip is a surgical emergency. The whole catheter must be removed aseptically in such a way that both contamination of the cerebral cavity and spillage into the peritoneum can be avoided. Awareness of this unusual complication among surgeons is needed for early recognition, management, and timely intervention to minimize morbidity.

## Background

The ventriculo-peritoneal shunt is used to drain the excess amount of cerebrospinal fluid in a unidirectional way from obstructed ventricular cavity into the peritoneum in the management of hydrocephalus. Ventriculo-peritoneal shunt sometimes causes complications. Bowel perforation caused by the ventriculo-peritoneal shunt is a rare occurrence with an estimated incidence rate of 0.1% to 1.0% among all cases of VP shunt displacement. Perforation of the bowel wall by VP shunt is a surgical emergency.

In the present case report, a child with spontaneous trans-anal extrusion of caudally migrated ventriculo-peritoneal shunt tip was managed with minimal surgical intervention. Literature was reviewed to find out therapeutic strategies.

## Case presentation

**Fig. 1:**
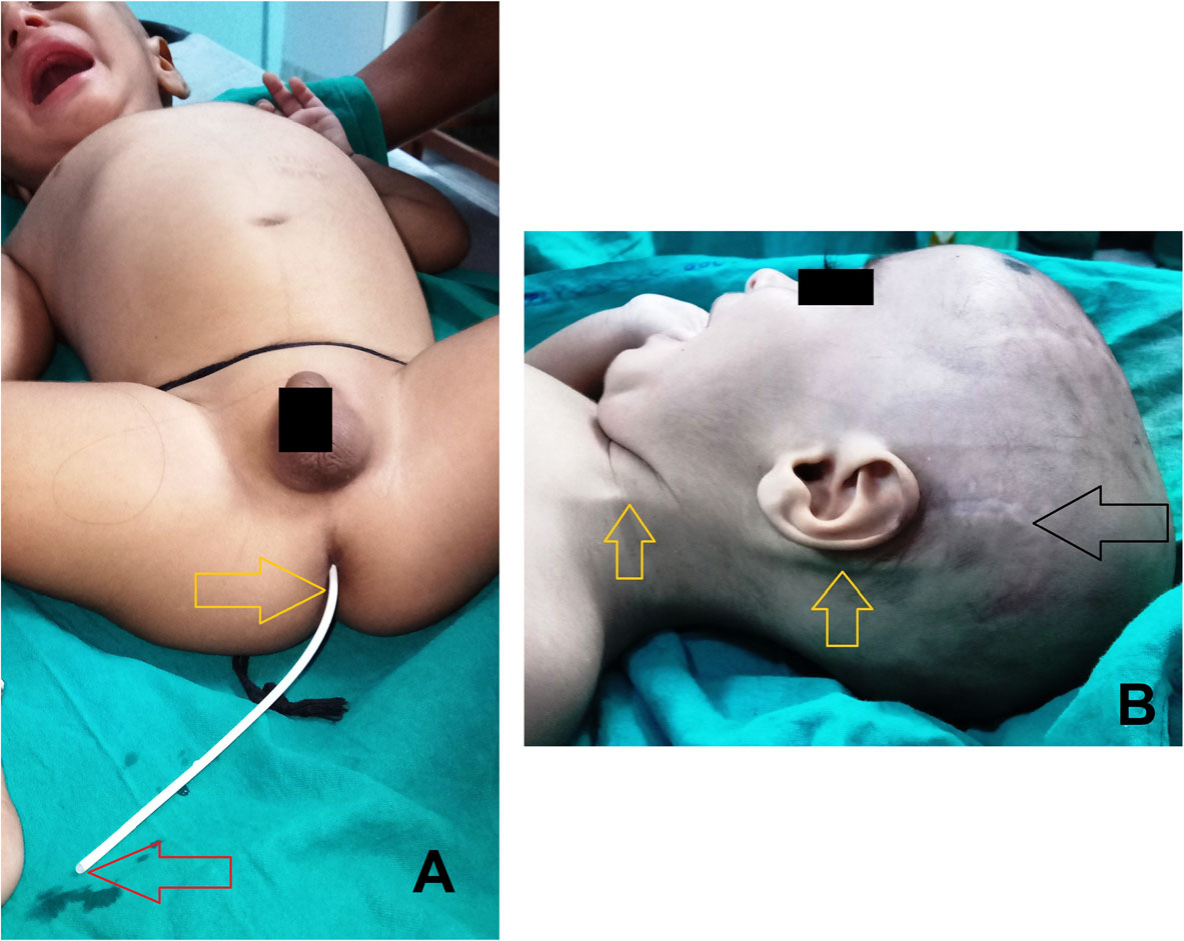
**a** Shows spontaneous trans-anal extrusion of caudally migrated distal part of the ventriculo-peritoneal shunt (yellow arrow) with functioning tip (red arrow, shows dribbling of CSF). **b** Shows previous operation scar mark in left temporal region (black arrow) with the position of VP shunt over the left neck region (yellow arrow)

**Fig. 2:**
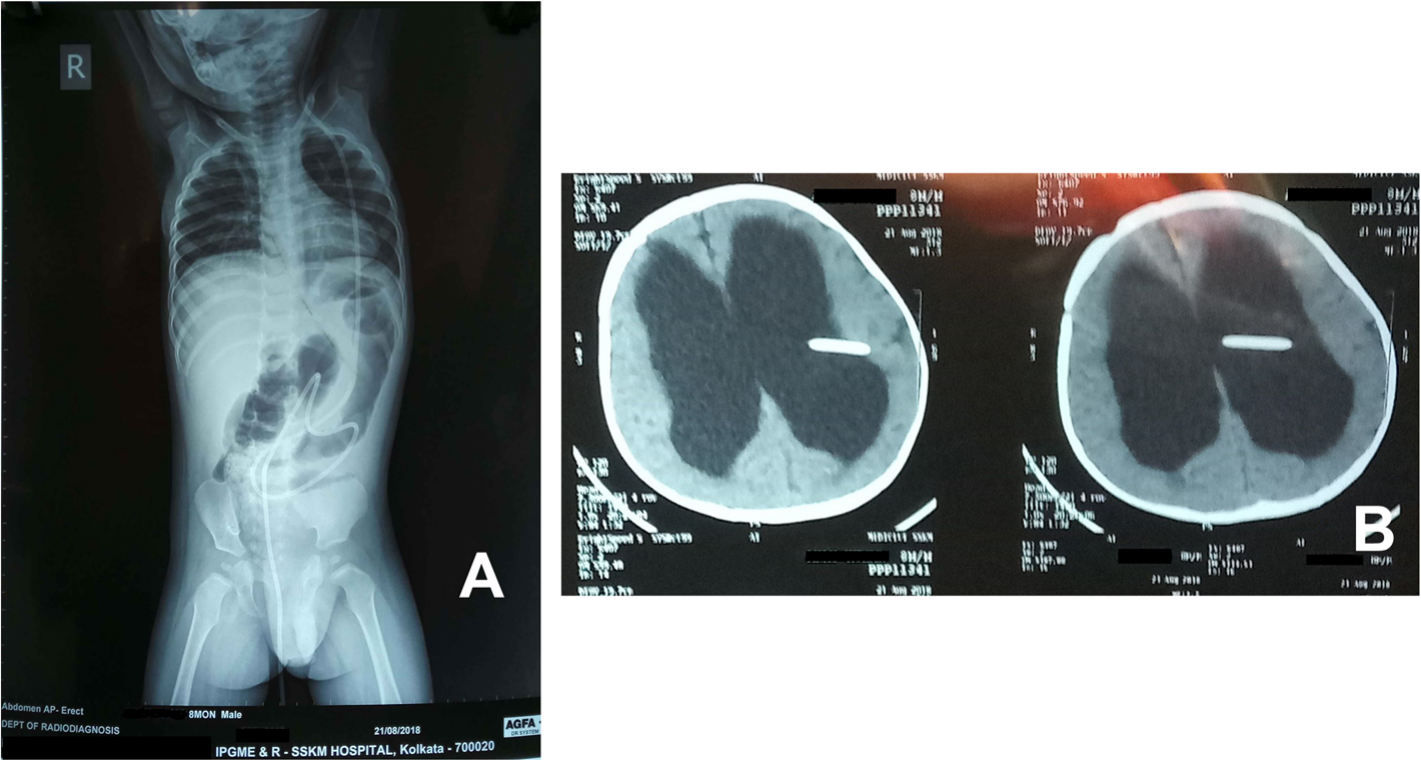
**a** Digital X-ray of whole abdomen postero-anterior view in erect posture shows expulsion of radio-opaque distal catheter tip through the anus into the exterior without any pneumoperitoneum. **b** NCCT scan shows the cranial catheter tip in the lateral ventricle of the brain

An asymptomatic 8 months old boy presented to the emergency department with spontaneous extrusion of ventriculo-peritoneal (VP) shunt per anus for last 1 day. Clear CSF was seen dribbling out through the distal tip of the catheter **(**Fig. 1a**)**. He had undergone Chhabra type silicone VP shunt placement surgery on the left side for congenital hydrocephalus 3 months ago (Fig. 1b, c). He had no fever, abdominal distention, vomiting or headache suggestive of peritonitis or encephalitis. Digital X-ray of the whole abdomen in postero-anterior view in erect posture was only evident of the expulsion of radio-opaque distal catheter tip through the anus into the exterior. No evidence of pneumoperitoneum or knotting of the shunt catheter was noted (Fig. 2a). Clinically no ascites could be demonstrated. Noncontrast-enhanced computed tomography brain scan (NCCT) showed a proximal catheter in the lateral ventricle of the brain (Fig. 2b, c).

Under sedation, the distal part of the VP shunt catheter was resected out, aseptically, over the abdomen and pulled out gently through the anus. The proximal catheter part along with the reservoir was removed through a separate incision in the neck and sent for bacteriological study, which came out later to be negative. Postoperatively, the child was put on a prophylactic antibiotic and resumed oral feed after 6 h. Three weeks after removal of the spontaneously extruded shunt repeat NCCT suggested persistence of hydrocephalus. So, another VP shunt was placed in the contralateral side.

## Discussion

Since the early 1900s, the diversion of cerebrospinal fluid (CSF) is established management for hydrocephalus [1]. The ventriculo-peritoneal shunt consists of various valve assemblies and a slit at its lower end common complications after ventriculo-peritoneal shunt placement is an infection of different parts of the central nervous system and formation of CSF fistula. The draining abdominal tip may also cause complications like paralytic ileus in the early postoperative period and intestinal obstruction secondary to adhesion formation in a later period. Some other abdominal complications may occur like blockade due to fibrous encasement or kinking, expulsion through the abdominal wound, migration (both cranial or caudal) into various abdominal organs [2], development of hydrocele in male, formation of peritoneal cyst and extrusion through the umbilicus [3, 4]. Perforation of the gall bladder, gut, rectal wall, urinary bladder, uterus, the vagina has also been reported in the literature [5].

Bowel perforation caused by VP shunt is extremely rare with an estimated incidence rate of 0.1% to 1.0% [6]. So far a total of around 112 reported cases of bowel perforation by VP shunt are available in the literature. Among the nearly half are in the age group of 0 to 10 years. Coexisting thin intestinal musculature in myelomeningocele, placement of hard peritoneal catheters, and local infective adhesions may predispose gut perforation [5].

The migration may be described in different ways, such as the following:

1. Based on direction— cranial or caudal migration.
2. Based on the location of migrated catheter tip—it may be total intracranial, subgaleal, migration in the breast, thoracic cavity, abdominal wall, in the hollow viscous or into the genitorurinary system.
3. Based on the migrated component of the shunt system—it may be proximal/distal catheter, valve, reservoir or entire shunt system migration.

On exploratory laparotomy or autopsy studies encasing fibrosis around the catheter tip on the bowel wall was found. So, it is hypothesized that the free beveled catheter tip gets adhered to the serosa [7] of the bowel wall giving constant pressure. Coupled with the local inflammatory reaction at that pressure point and continuous water hammer effect of the CSF pulsations, gut wall perforates. Thereafter, the freely moving catheter tip is driven forward by peristalsis and eventually brought out through the anus [8]. Patients may present with significant abdominal symptoms or peritonitis in less than 25% cases [8]. Surgeons should be aware of this rare condition to avoid potential morbidity arising from an infection like meningitis, ventriculitis, and sepsis [8] which may be associated with mortality rate up to 15% [9].

During the management of this unusual complication, features of ascending gram-negative bacterial meningitis, sepsis, perforative peritonitis, or intraperitoneal abscess formation should be considered individually. The decision for optimum treatment of such a patient should be taken accordingly. In a patient with intestinal perforation but no other complications, a formal exploratory laparotomy is not required. The treatment for acute (within few days) cases with peritonitis may need an emergency exploratory laparotomy with shunt removal, thorough peritoneal lavage and primary repair of the intestinal wall [10]. But in chronic cases (after weeks/months) only detachment of shunt catheter at the abdominal wall and removal of the distal end through the anus (rarely colonoscopy/sigmoidoscopy guided) is only needed. The cut distal end is not pulled back to avoid contamination of the tract. External ventriculostomy (using proximal part of the shunt system) may be maintained at least for some weeks after putting the patient on broad-spectrum antibiotics. If repeated bacteriological culture of CSF comes negative then VP shunt placement on the opposite side should be performed.

Sometimes, knotting of long shunt catheter itself or twisting of the tube with the bowel loops makes laparotomy mandatory even in the absence of peritonitis. Alternatively, laparoscopic visualization and disengagement of the shunt tube may be tried. Simultaneous laparoscopic management of intra-abdominal complications along with endoscopic management of anal extrusion of ventriculo-peritoneal shunt has also been reported in the literature [11].

## Conclusions

Spontaneous trans-anal extrusion of caudally migrated ventriculo-peritoneal shunt tip is a surgical emergency. The whole catheter must be removed aseptically in such a way that both contamination of the cerebral cavity and spillage into the peritoneum can be avoided. External ventricular drainage may also be needed under proper antibiotic coverage and once the CSF bacteriological cultures are negative, a new peritoneal shunt should be placed contralaterally.

In absence of peritonitis or abdominal abscess, manual removal of distal part through anus after resection over abdominal wall should be done. In the case of knotting/adhesions, laparoscopic or endoscopic removal of the catheter may be tried. The fibrous tissue on the gut wall at the perforation site prevents the spillage of bowel contents into the peritoneum. Laparotomy must be performed in cases of peritonitis or intra-abdominal abscess formation or when the spontaneous closure of the fistulous tract fails after percutaneous or endoscopic removal.

Awareness of this unusual complication among surgeons is needed for early recognition, management, and timely intervention to minimize morbidity.

## Data Availability

Presented within the manuscript, please contact the author for additional data requests.

## Abbreviations

CSF: Cerebrospinal fluid
NCCT: Noncontrast-enhanced computed tomography
VP: Ventriculo-peritoneal
